# The Value of Iridectomy in Blindness, Resulting from Opaque Cornea

**Published:** 1898-11

**Authors:** A. Bethune Patterson

**Affiliations:** Atlanta, Ga.


### THE VALUE OF IRIDECTOMY IN BLINDNESS, RESULTING FROM OPAQUE CORNEA.

BY A. BETHUNE PATTERSON, M.D.,
ATLANTA, GA.

The following cases are selected from a number of similar ones, illustrative of the value of iridectomy in cases of blindness, which have resulted from opacity of cornea, produced by ulcerative keratitis (corneal ulcers). Only the worst forms will be included in this report, where the healthy corneal tissue is nearly supplanted by the formation of opaque cicatricial tissue.

There are several forms of ulceration, which have a tendency to bring about this condition of the cornea.

J. W., aged thirty; two years previous to his coming had a severe form of inflammation which left him blind, from the conversion of the once transparent cornea into a white cicatricial membrane, complete in right eye, so that there was not the slightest bit of iris visible, staphylomatous and with history of recurrent ulcerations. I lost no time in enucleating this contracted and chronically inflamed eye.

The cornea of left eye was as white as right, more perfect in outlines, and free of tenderness and hardness. At the lower margin a transparent cornea, possibly a little more than a millimeter in width, and in length about one-third of its circumference.

Three months after enucleating the right eye, the left conjunctival sac was daily irrigated with an antiseptic lotion, and an antiseptic dressing applied, until the time appointed for the operation. The iridectomy was done under thorough antiseptic precautions. There was no inflammatory action in the track of incision, and, therefore, no impairment to the transparent strip of cornea. Through this narrow slot, this unfortunate and unhappy man was given useful vision, which enabled him to get about without assistance, and was enabled to recognize familiar faces at about fifteen paces. As he did not know his letters, no accurate estimate of vision could be made.

The last account I had of this case was five years after the operation; at that time there had been no diminution of vision. He was making himself useful in attending to stock and assisting in work that did not require accurate eyesight.

S. P., aged nine; has been blind from infancy; history of ophthalmia neonatorum. Both cornea was a dense mass of white cicatricial tissue; could only distinguish daylight from darkness. The right eye presented a margin of transparent cornea below, or at its lower margin; its greatest width would not exceed two millimeters. In left eye there was a transparent line of cornea occupying the upper and outer quadrant, showing not more than half as much of the dark iris as its fellow eye; the latter I selected as the one best suited for an iridectomy.

The eye was prepared for operation as follows: A lotion of boracic acid, fifteen grains to the ounce of water, was used every few days to irrigate the conjunctival sac, keeping the eye bandaged with antiseptic gauze and cotton.

The iridectomy was done while under the influenc of chloroform, as I could not afford to take any risk with cocain, as with it there is always a sharp pain experienced when the iris is seized and excised; therefore, I have preferred chloroform anesthesia.

The iridectomy proved a success; it gave the child useful vision. I am unable to state the amount, as I have not seen the patient since his recovery from operation, soon after which he was removed to his home in an adjoining State. The last account I had of him was three years after the operation; there had been no deterioration in vision, as well as mother could judge; he was making some headway at spelling.

C. D., aged twenty; dates his blindness from infancy; both cornea opaque; only the right afforded any encouragement for operative interference, and really I entertained very little hope of an iridectomy doing much for him, as the point at which the brown iris could be seen was semi-transparent.

For ten days the eye was kept bandaged and irrigated every other day with a saturated solution of boracic acid.

On the morning of the operation the eye was cocainized and the iridectomy made. I was careful in getting away a good bit of iris. There was no inflammatory reaction, and the eye soon recovered from the operation. I kept this case under observation for six weeks; at the time of his leaving could count fingers with some difficulty at ten paces; this amount of sight enabled him to get about without assistance, and made him a more useful man to himself and others.

While serving as assistant in the Royal London Ophthalmic Hospital, I on one occasion drew Mr. Nettleship's attention to a patient whose eye was in a similar condition to number one in the above report, and asked what he thought of the chances of an iridectomy. He replied that he did not think favorably of the success of such an operation, that the good effects did not last very long, as the transparent cornea was soon invaded by gray infiltration from the inscision in the sclero-corneal junction. I believe my success in preventing this infiltration was due to the thorough antisepsis in the toilet of operation, and also, especially to the preparation the eye underwent in rendering it as aseptic as possible prior to the operation.

From my experience in hospital and private practice, I am convinced that a very large per cent. of blindness from extensive leucomas and cicatricial tissue have their origin in gonorrheal ophthalmia; as is well known, this is a disease extremely fatal to sight, especially when neglected at the onset.

In ophthalmia neonatorum the first evidences of the disease is observed about the third or fourth day after birth. As a prophylactic in this serious complaint, I would urge upon the attention of the accoucheur the importance of making inquiry into the vaginal secretion, whether or not there is or has been a leucorrheal discharge or whites; where there is the slightest suspicion of such unhealthfulness, no time should be lost in repeated thorough irrigations of the vagina with antiseptic lotions, thus lessening the chances of the child's eyes becoming affected from the vaginal secretion. I cannot lay too much stress upon the immediate cleansing and disinfecting the eyes. It would be a safe and wise precaution immediately after the first cleansing, to instill into the eyes a drop of a solution of nitrate of silver, ten grains to the ounce of water, followed by a weak solution of salt and water to neutralize the nitrate of silver. No possible injury can result from this treatment to the eyes of the new-born; it would be almost a sure preventive against infection.

The cleansing and disinfection of the eyes of the infant should be done by the physician, and not entrusted to the nurse.

*507 English-American Building.*

---

L. FREYBERGER (*Treatment*, May 12, 1898) has treated thirty cases of incontinence of urine with fluid extract of rhus aromatica, with permanent cure in eighteen and with relief in ten others. The dose employed was from five to ten minims three times a day for children from two to five years old, and from ten to fifteen minims for children from five to ten. The disagreeable taste can be disguised by aromatic sirup.

